# Objective structured clinical examination versus traditional written examinations: a prospective observational study

**DOI:** 10.1186/s12909-023-04050-5

**Published:** 2023-01-28

**Authors:** Souhil Lebdai, Béatrice Bouvard, Ludovic Martin, Cédric Annweiler, Nicolas Lerolle, Emmanuel Rineau

**Affiliations:** 1 Urology Department, University Hospital of Angers, 49933 Angers cedex 9 Angers, France; 2 Health Faculty, University of Angers, Angers, France; 3 All’Sims Center for Simulation in Healthcare, University Hospital of Angers, Angers, France; 4 Department of Anesthesia and Critical Care, University Hospital of Angers, Angers, France

**Keywords:** Medical education research, Simulation, OSCE, Clinical skills, Patient management

## Abstract

**Background:**

Recently, Objective Structured Clinical Examinations (OSCE) became an official evaluation modality for 6-year medical students in France. Before, standard examination modalities were: written progressive clinical cases (PCC), written critical reading of scientific articles (CRA), and internship evaluation (IE). The aim of this study was to assess the performances of 6-year medical students in their final faculty tests by comparing OSCE-exams with standard examination modalities.

**Methods:**

This was a prospective observational study. We included all 6-year medical students in our university from 2020 to 2021. The endpoints were the scores obtained at the following final faculty tests during the 6^th^ year of medical studies: OSCE-training, OSCE-exams, written PCC, written CRA, and IE. All scores were compared in a paired-analysis.

**Results:**

A total of 400 students were included in the study. No student was excluded in the final analysis. The mean scores obtained at the OSCE-exams were significantly different from those obtained at OSCE-training, PCC, CRA, and IE (12.6 ± 1.7, 11.7 ± 1.7, 13.4 ± 1.4, 13.2 ± 1.5, 14.7 ± 0.9, respectively; *p* < 0.001). OSCE-exams scores were moderately and significantly correlated with OSCE-training and PCC (Spearman rho coefficient = 0.4, *p* < 0.001); OSCE examination scores were lowly but significantly correlated with CRA and IE (Spearman rho coefficient = 0.3, *p* < 0.001). OSCE-scores significantly increased after an OSCE training session.

**Conclusion:**

In our faculty, 6-year medical students obtained lower scores at OSCE exams compared to other standard evaluation modalities. The correlation was weak to moderate but significant. These results suggest that OSCE are not redundant with the other evaluation modalities. Interestingly, a single OSCE training session led to an improvement in OSCE scores underlining the importance of a specific training.

## Introduction

Problem-based learning and clinical simulation have been playing an increasingly important role in contemporary medical training [1–8]. For many years, objective structured clinical examination (OSCE) has shown its effectiveness in assessing medical students, and is already commonly used in many countries such as the United States, Canada, Australia or India; however, its integration in national medical training programs has been quite recent in many countries, especially in Europe [3, 9–11].

OSCE involve most of the time short simulated clinical scenarios in order to test various skills such as interviewing, clinical reasoning, data interpretation, clinical examination and management strategies. In France, the 6-year medical students must validate a certificate of “clinical competence”, which is mandatory before starting their residency. Validation modalities for this certificate have been recently modified by adding OSCE-exams to the standard examinations which include written progressive clinical cases (PCC), written critical reading of scientific articles (CRA), and internship evaluation (IE). In our faculty, the OSCE-exams include 9 consecutive 7-min stations, covering various areas of medical expertise: technical skills, patient interview, patient education and prevention, imaging interpretation, delivering medical news, diagnostic strategy, clinical examination, interpretation of paraclinical tests results, and patient management strategy.

Interestingly, it is not certain that succeeding in OSCE-exams is correlated with succeeding in other assessment modalities (such as long case examination, MCQ, essays, oral exams), as shown by the contradictory results of several studies [10–17]. Most of these studies report correlation coefficients ranging from 0.1 to 0.6. In addition, it is also uncertain if prior specific training is required before OSCE-exams [18]. These questions are critical because of the complex organization of these large-scale OSCE tests which require major logistical and human resources, either for official exams or for training [3, 19, 20]. Addressing these questions might indeed justify the necessity of a specific training for OSCE and for clinical skills in general, in addition to regular academic training. The aim of this study was to assess the performances of 6-year medical students in their final faculty tests by comparing scores obtained at OSCE-exams with standard examination modalities. The secondary objectives were to determine whether or not OSCE scores were correlated with other examination modalities, and to determine if OSCE scores improved after training.

## Methods

### Participants

This prospective observational study was performed in the Health Faculty of the University of Angers, in France.

We included all 6-year medical students from our faculty during from March 2020 to December 2021. This study was carried-out in accordance with French regulations. The approval of an ethics committee or the consent of the participants was not required as this study was not involving the Human person according to the French Public Health Code (*Loi Jardé—n°2012–300 of March the 5*^*th*^* 2012, in application in November 2016—Article R1121-1: research conducted exclusively from the processing of personal data is outside the scope of the RIPH*).

### Examination methods

#### OSCE

All OSCE were created based on the French National OSCE guidelines [21]. Students underwent 2 OSCE sessions: one training session and one evaluation session, 6 months apart (one in March and one in December). In our Faculty, OSCE training consisted in OSCE sessions with the exact same set-up, but with different cases. Evaluation modalities were identical in both sessions (described below). Our objective was to train the students to the OSCE examination modality in order the suppress the performance bias due to the fact that none of the students had been exposed to OSCE before. Each session consisted of 9 consecutive 7-min OSCE stations, with a one-minute pause between each OSCE. The OSCE-exams covered several areas of expertise, as required by the national program: technical skills, patient interview, patient education and prevention, imaging interpretation, delivering of medical news, diagnostic strategy, clinical examination, interpretation of paraclinical tests results, patient management strategy. The OSCE could cover any medical specialty. The evaluation was performed by two independent academic examiners who individually scored the students’ performance using a check-list evaluation grid validated by the university OSCE faculty. For each student, the average score for each of the 9 OSCE stations was calculated and the final OSCE-exam score was the average score of the 9 OSCE stations.

Each OSCE sessions set-ups were standardized and reproductible. No OSCE had been carried-out for these students before inclusion in 2020. About 100 teachers were involved for each OSCE session. In order to control the evaluation bias, all teachers followed the same training for OSCE evaluations and all the OSCE were reviewed by an independent committee in order the have a standardized evaluation process in accordance to the French OSCE guidelines [21].

#### Other examination methods

During the same 6-month period, students also underwent “regular” examinations in the form of: independent MCQs tests, progressive clinical cases (including 10 to 15 MCQs for each test) and critical reading of a scientific article (including 2 MCQs tests). They also obtained an internship evaluation score, which was the mean score of all of their hospital internships evaluations during the past 4 years. As for OSCEs, there were no differences in organization for these evaluation methods between the years 2020 and 2021.

### Objectives, endpoints and definitions

Scores obtained by students were compared in a paired analysis: each student was his own comparator. Each student had 5 scores: the OSCE-training score, the OSCE-exam score, the PCC score, the CRA score, and the IE score. The scores ranged from 0 to 20 for all types of examination, in accordance with usual standard scoring and evaluation procedures in France. A very good grade corresponded to score between 15 and 20, an average grade to a score between 10 and 14.9, and a low grade corresponded to a score between 0 and 9.9.

The main objective was to assess whether the addition of OSCEs brought added value to the usual evaluation methods of our faculty. To answer this question, several criteria were evaluated: the comparison of scores between the different types of examination, the comparison of their standard deviations, and the correlation between the scores obtained in the OSCE-exams and each of the other evaluation modality. In addition, a multivariate analysis assessing the chance of obtaining a grade higher than 10 or higher than the median score of all the students was carried-out.

The secondary objective was to assess whether carrying-out an OSCE training provided added value compared to an OSCE evaluation alone. In order to answer this question, the scores obtained at the OSCE-training and OSCE-exams were compared. In addition, an assessment of the grade’s trajectories between these 2 OSCE sessions was performed.

### Statistical analysis

Statistical analysis was conducted using SPSS 15.0 Software® (IBM Corp., Armonk, NY, USA). Means are reported with standard deviation (SD), medians are reported with range. Qualitative variables were compared using a Chi-square test. Paired quantitative analysis was conducted using a Wilcoxon test in order to compare the scores obtained by each student according to the different types of tests. Unpaired quantitative analysis was conducted using a Wilcoxon test in order to compare the global scores according to the different types of tests. The Spearman rank correlation test was used to assess the correlation between the scores according to the different types of tests. A logistic regression was used for multivariable analyses, all types of tests were included in the model as all of them were significantly associated with the assessed effect in univariate analysis. Internal consistency was assessed with the Cronbach alpha coefficient. Statistical significance was defined as p < 0.05.

## Results

### Population and internal consistency of the examinations

**Fig. 1:**
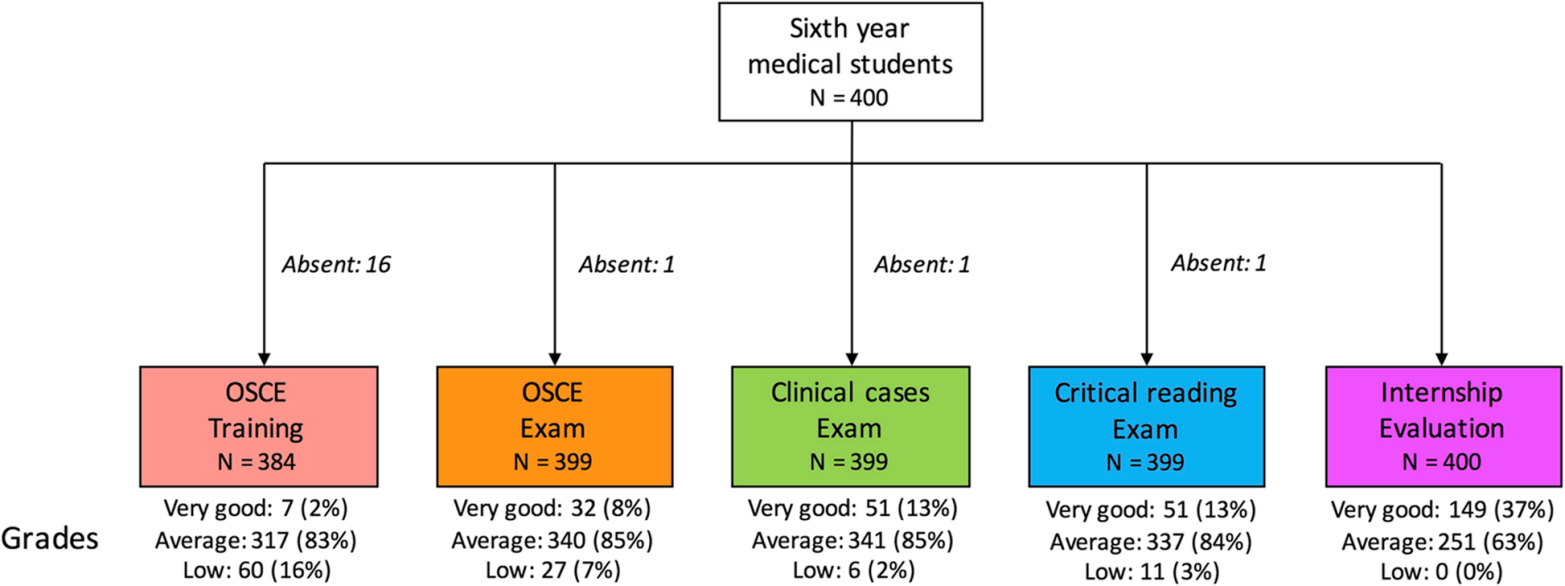
Flow chart of the study

We included a total of 400 students in the study (222 in 2020 and 178 in 2021): 274 (68.5%) women and 126 (31.5%) men. There were no significant differences in scores between both years for each examination category (OSCE-training, OSCE-exam, PCC, CRA and IE). The flow chart of the study is presented in Fig. 1. Sixteen students were absent for the OSCE-training, but only one student was absent for the OSCE, PCC and CRA examinations.

The Cronbach alpha coefficients for the whole exam session (OSCE, PCC and CRA) and for OSCE specifically were 0.7 and 0.6, respectively.

### Comparison of scores obtained in all exams

**Fig. 2:**
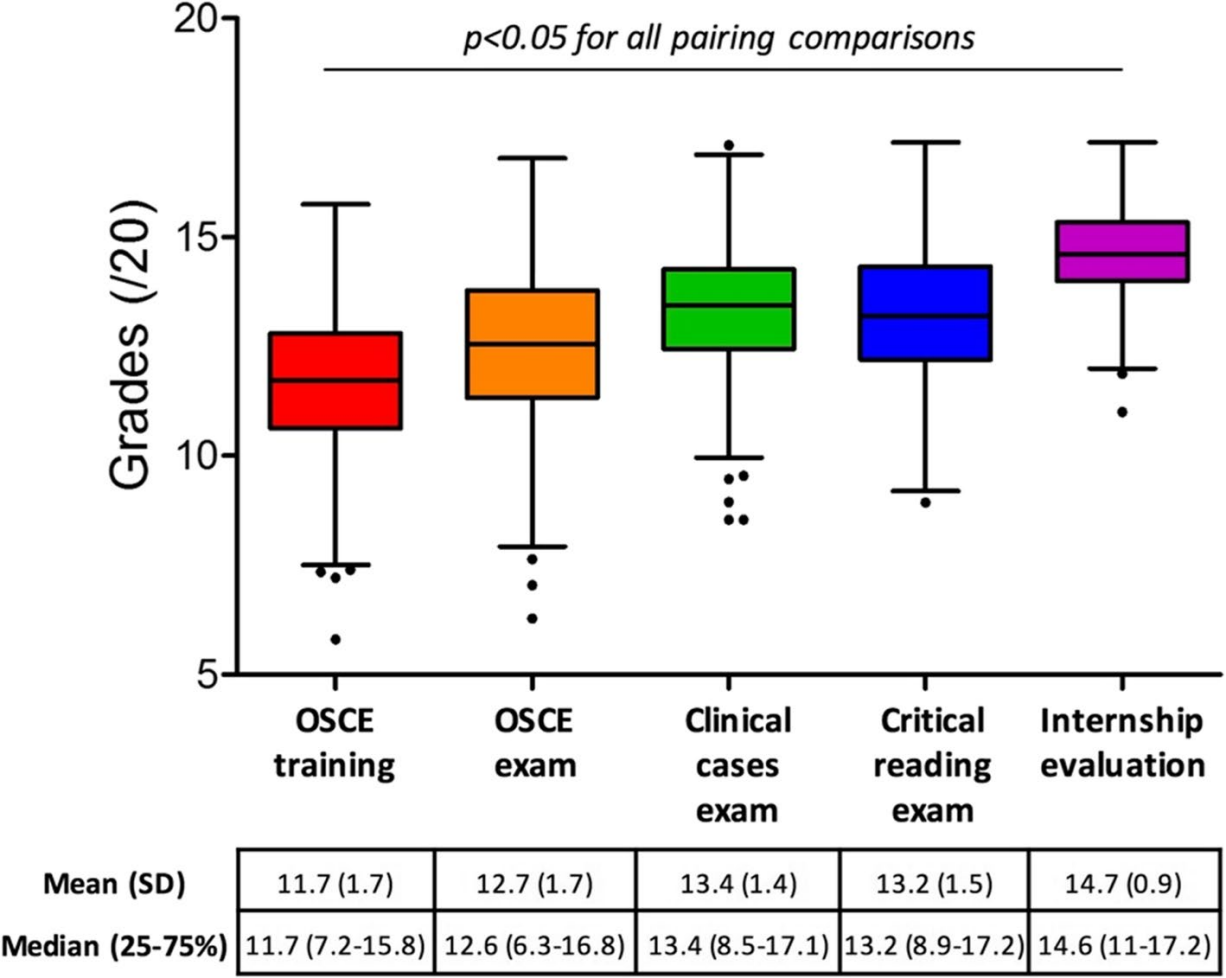
Grades obtained at the OSCE training (red), OSCE exam (orange), clinical cases exam (green), critical reading exam (blue) and at the internship evaluation (purple). Graphs are boxplots with medians, Tukey whiskers and points for outlier values. Means with standard deviations (SD) are also presented for each exam type in the table below the x-axis and compared in a paired analysis. In paired analysis, *p* values were < 0.001 for comparisons of grades between OSCE training and OSCE exam, OSCE training and Clinical cases exam, OSCE training and Critical reading exam, OSCE training and Internship evaluation, OSCE exam and Clinical cases exam, OSCE exam and Critical reading exam, OSCE exam and Internship evaluation, Clinical cases exam and Internship evaluation, and Critical reading exam and Internship evaluation, and *p* value was 0.033 for the comparison of grades between Clinical cases exam and Critical reading exam

For the whole population, the mean OSCE examination grade was 12.6 (SD 1.7). Scores obtained for each examination type are presented in the Fig. 2. Mean scores of the whole population were above 10 for all examination types. The lowest mean scores were those obtained at the OSCE-training followed by the OSCE-exams, and the best mean score was that of the internship evaluations. In paired analysis, scores were all significantly different from each other between all types of tests (*p* < 0.05, details in Fig. 2). In unpaired analysis, scores were all significantly different from each other (*p* < 0.001) except for PCC versus CRA (*p* = 0.107). The standard deviation was significantly higher for the OSCE-exams, in comparison to PCC, CRA and IE (*p *< 0.001).

### Correlations between the OSCE grades and the other exams grades

**Fig. 3:**
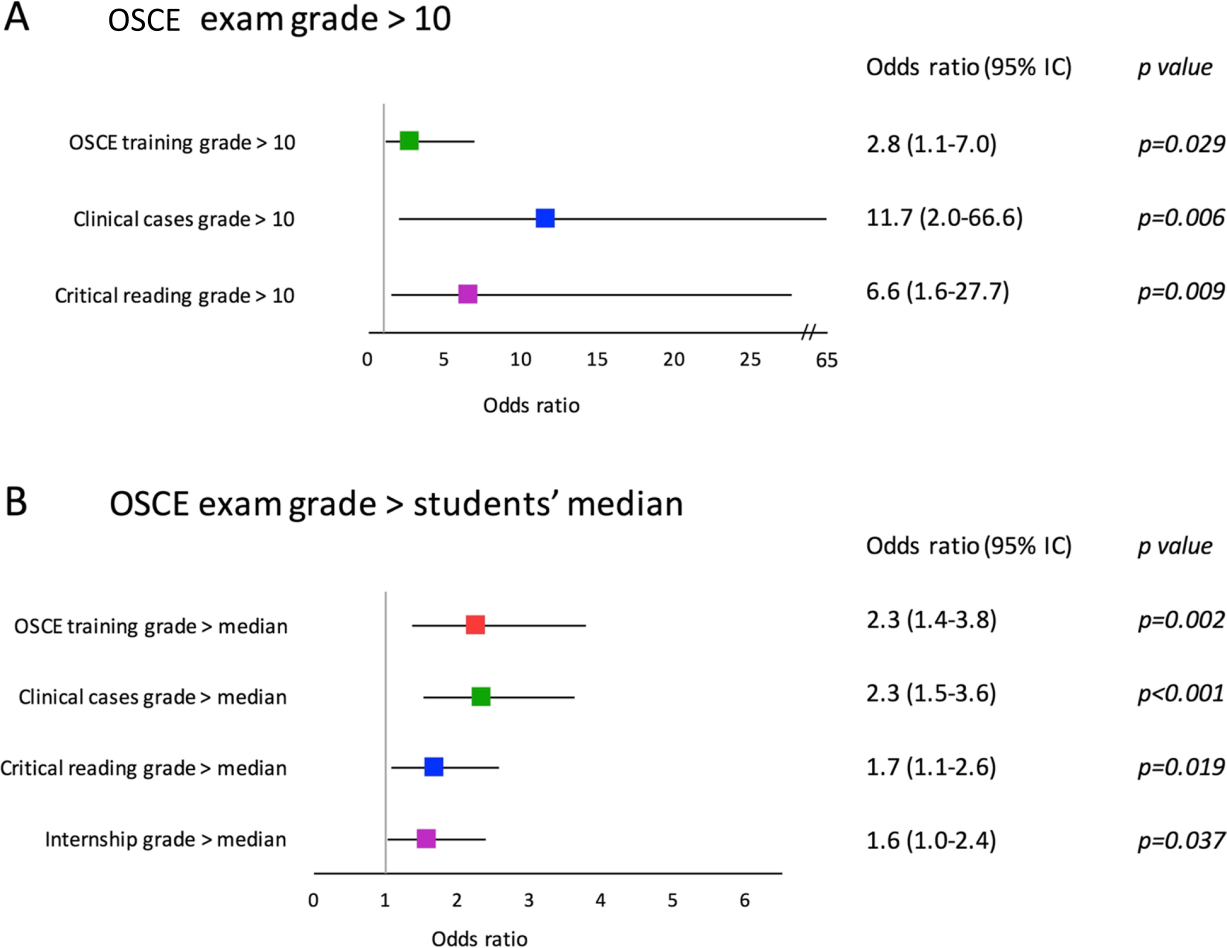
Forrest plot representing odds-ratios with their confidence intervals at 95%. **A** multivariate analysis assessing the chance to obtained a grade > 10 at the OSCE exam. **B** multivariate analysis assessing the chance to obtain a grade > median at the OSCE exam. Medians correspond to the medians of the grades obtained for each type of examination by all students

We examined in univariate and multivariate analysis the influence of having scores > 10/20 or above the median score for the OSCE training, the PCC, the CRA and the IE on the chances of having an OSCE-exam score > 10/20 or above the median score. Results of the univariate analysis are presented in Table 1 and results of the multivariate analysis are presented in Fig. 3. Grades > 10 for OSCE training, PCC, and CRA were independent factors for an OSCE-exam score > 10/20 or above the median score, and a grade above the median for internship evaluation was an independent factor for an OSCE examination grade above the median.

**Table 1:** Univariate analysis for having an OSCE score superior to 10/20 or above the students’ median

Studied parameter	Odds ratio	95% IC inferior	95% IC superior	p
For OSCE score > 10				
OSCE training score > 10	3.800	1.647	8.767	0.003
Clinical cases score > 10	16.087	3.075	84.168	0.004
Critical reading score > 10	5.951	1.479	23.945	0.029
Internship score > 10	NA	NA	NA	NA
For OSCE score > median score				
OSCE training score > median	3.102	1.912	5.03	< 0.001
Clinical cases score > median	3.229	2.145	4.86	< 0.001
Critical reading score > median	2.249	1.507	3.356	< 0.001
Internship score > median	1.906	1.281	2.835	0.001

*OSCE* Objective Structured Clinical Examination, *OR* Odds ratio, *IC95%* 95% interval of confidence, *NA* not applicable

**Fig. 4:**
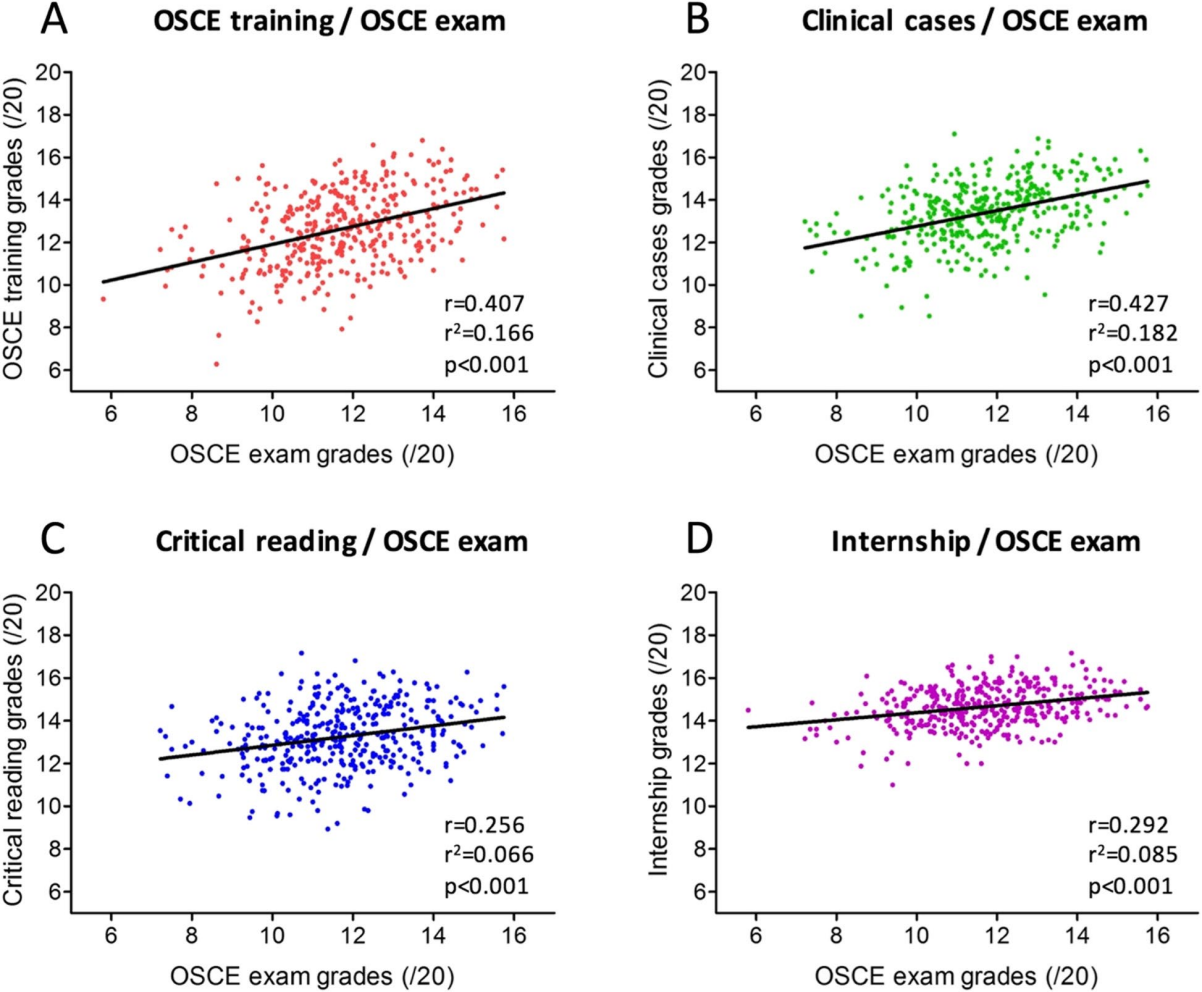
Correlations between the grades obtained at the OSCE exam and the OSCE training (**A**, red), clinical cases exam (**B**, green), critical reading exam (**C**, blue) and the internship evaluations (**D**, purple). The letter r represents the Spearman rank correlation coefficient

OSCE-exams scores were moderately and significantly correlated with OSCE-training and PCC (Spearman rho coefficient = 0.4, *p* < 0.001) (Fig. 4A and B); OSCE examination scores were lowly but significantly correlated with CRA and IE (Spearman rho coefficient = 0.3, *p* < 0.001) (Fig. 4C and D).

### Comparison between grades obtained at the OSCE-training and at the OSCE-exams

**Fig. 5:**
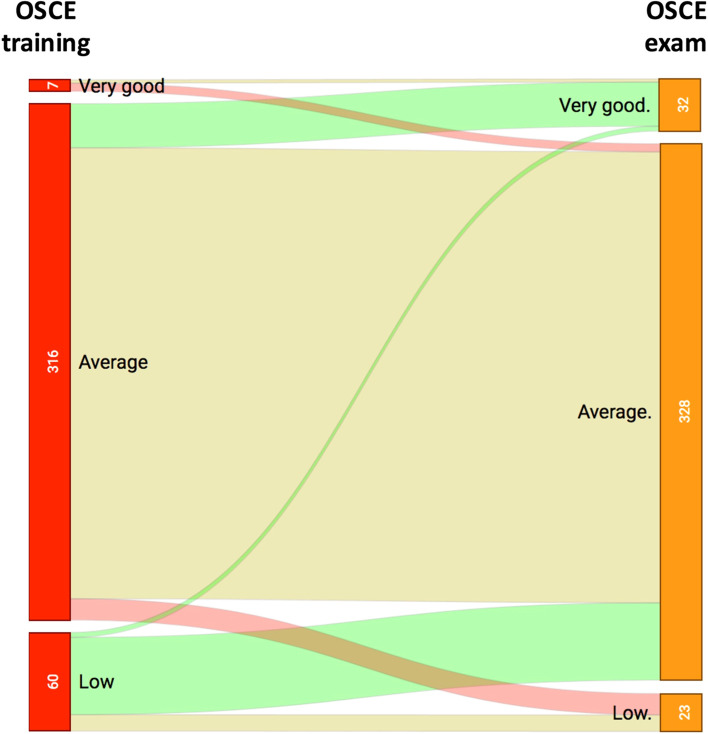
Trajectories of grade levels between the OSCE training (red) and the OSCE exam (orange). The red-light trajectories show a decrease, the green ones an improvement and the beige ones a stability. The numbers are the numbers of students. The very good, average and low levels correspond to grade between 15–20, 10–14.9 and 0–9.9 respectively, on a grading scale between 0 and 20

As shown in Fig. 5, the grades obtained at the OSCE-exams were significantly higher than in the OSCE-training (paired and unpaired tests). Figure 5 illustrates the evolution of students’ performances between OSCE training and exams. Students’ trajectories are presented according to their score categories between OSCE training and exams, and show students’ repartition and evolution between both sessions. A majority of students (316 (83%)) had an average grade-level during the training session, and a majority of them kept this grade-level during the exams (276 (87%)). However, 77 (20%) students improved their level of performance during the exams, while 18 (5%) downgraded the performance (*p* < 0.01).

## Discussion

Performing OSCE was a national decision applied to all medical schools in France. The aim of this study was to analyze real life data and compare OSCE scores to previous historical evaluation modalities. In this study, we observed that 6-year medical students obtained lower scores at the OSCE-exams compared to the other standard evaluation modalities in a paired analysis. The correlation between the OSCE-exams and the other evaluation modalities was weak to moderate but still significant. These results suggest that OSCE may not be redundant with the other evaluation modalities and might improve students’ evaluation.

One of the major objectives of the OSCE in medical learning is to assess the medical skills required to become a good physician. The assessment of these skills is difficult to achieve and requires either a real or a simulated clinical situation. These situations may include technical skills (lumbar puncture, urinary catheterization, etc.) or non-technical skills (patient interviewing, information delivery, interprofessional communication) which were traditionally assessed with written evaluations (MCQs for example), or during real-life clinical evaluation in the presence of a patient and a practitioner. The major development of medical simulation in universities over the past 10 years has probably helped to make this kind of evaluation more easily accessible. Medical students therefore have more regular access to medical simulation during their first 6 years of medical school. However, the organization of large-scale OSCE examinations for several hundreds of students at a time requires major logistical and human resources [3]. This raises the question of the real added value of these evaluations. Matet et al. showed that OSCE increased the discriminatory capacity of current evaluations modalities in French medical schools [11]. Furthermore, Pugh et al. showed a correlation ranging from 0.305 (*p* = 0.002) to 0.516 (*p* < 0.001) between scores from an OSCE progress test and the written component of the national high-stakes examination [22]. Furthermore, in a study by Tijani et al., OSCE and long case examination had a correlation of 0.374 and, compared with the long case examination, the OSCE had a higher correlation with all other forms of assessment (MCQ and essays) [16].

In our study, we observed a significant correlation between the OSCE scores and the other types of tests, which could suggest at first sight a redundancy for students’ evaluation. However, with a closer look, we first noticed that this significant correlation was weak to moderate, which means that having a high score with one modality did not necessarily imply a high score with the others. Secondly, the score distribution was significantly larger with the OSCE when compared to the other evaluation modalities, making OSCE more discriminant, especially when compared to internship evaluation which was traditionally the main clinical skill evaluation method in the past. Finally, the OSCE scores were significantly lower than those obtained with the other types of examination. Interestingly, in the study of Matet et al., this correlation was even weaker although significant, but the OSCE-scores were higher than those obtained at MCQ-based examinations [11]. Other studies also reported similar findings with a correlation coefficient between OSCE and MCQ ranging from 0.3 to 0.4 [12, 16]. In our opinion, these results suggest that OSCE brings and added value to MCQs and other traditional examination modalities by evaluating a large variety of skills and in a different way: for instance, MCQs assess mainly medical knowledge and have little ability to assess clinical skills [23]. Consistently with other studies we observed a significantly larger distribution of grades obtained at OSCE compared to grades from other academic evaluation modalities [11]. This underlies the potential discriminating power of OSCE for student ranking. In our study, the 9 stations assessed 9 different skills and it would probably be useful in the future to assess whether certain types of stations are even more discriminant than others.

Regarding the scores obtained in our study, we found that having a score above the median score or above 10/20 in each of the usual examination modalities were independent predictive factors of having an OSCE examination-score above the median score or above 10/20. This shows that even if the scores were different from an examination modality to another, a majority of students who managed to have a grade above the median score had a significantly higher chance to perform equally in the other examinations. This fact is reinsuring because it means that despite the differences between scores, a student capable of validating one modality with a score > 10/20 is likely to validate the others.

In addition, we observed that the OSCE examination scores were significantly higher than the OSCE training scores that took place 6 months before. This result is a major point that demonstrates the immediate progression of students after only one training session in real conditions. It is worth-mentioning that in this study we were able to include 400 students totally naïve from OSCE and make them undergo real-life OSCE-training in the exact same conditions as the OSCE-exams. This was a unique situation in our center because it is now mandatory to have at least one OSCE training session during all internships in each medical or surgical department. Showing a progression between the two OSCE sessions in naïve students demonstrated the impact of training in real conditions. These results are frequently found in studies evaluating the skills of students or doctors in the context of medical simulation [18, 24, 25]. OSCE scores were found to have high reliability and demonstrated significant differences in performance by year of training, providing evidence for the validity of OSCE scores as markers of progress in learners at different levels of training [26]. In our study, the training session and the actual OSCE examination had an identical set-up but the questions were different. Therefore, the better performance might be related to a better comprehension of the OSCE modality in general. But on the other hand, having different cases and questions is also a potential bias. We however believe that the main reason for the better performance of the students is that most of them understood better the OSCE modality and could use their time more efficiently and with less stress. All this should be assessed and proven in further studies.

This study has several limitations, some of which have been already been discussed above. Another important bias was that this study did not have a control group. It would have been interesting to see if the scores obtained at the OSCE examination session would have been as good without any OSCE training session 6 months earlier. However, in this study, each student was his own control and we therefor were able to assess evolution of their performances and to compare their OSCE scores with the other types of examinations. There were about 100 evaluators involved during the examination sessions, but all had the same training for OSCE creation and evaluation and, importantly, each OSCE was scored using an accurate and standardized grid that was centrally validated by the OSCE referent teachers.

To our knowledge, this OSCE study is among the largest series published with 400 participating students. In our opinion, this study justifies the necessity of a specific large-scale and systematic OSCE training for clinical skill acquisition in addition to regular academic training.

## Conclusion

In our faculty, 6-year medical students obtained lower scores at OSCE exams compared to other standard evaluation modalities with a significantly wider range. The correlation between OSCE and other modalities was weak to moderate but significant. These results suggest that OSCE are not redundant with the other evaluation modalities and confirm the inextricable links between theoretical knowledge and the practical application of this knowledge. Interestingly, a single OSCE training led to an improvement in OSCE scores 6 months later. These outcomes underline the importance of implementing this type of training and evaluation during medical curriculums in a broader way. Nevertheless, OSCE implementation in faculties is a challenge that needs a specific and dedicated training program for both medical students and lecturers.

## Data Availability

The datasets used and/or analyzed during the current study are available from the corresponding author on reasonable request.

## Abbreviations

CRA: Critical reading of scientific articles
IE: Internship evaluation
MCQ: Multiple-choice questions
PCC: Progressive clinical cases
OSCE: Objective structured clinical examination
SD: Standard deviation
